# Improving needle biopsy accuracy in small renal mass using tumor-specific DNA methylation markers

**DOI:** 10.18632/oncotarget.12276

**Published:** 2016-09-27

**Authors:** Sameer Chopra, Jie Liu, Mehrdad Alemozaffar, Peter W. Nichols, Manju Aron, Daniel J. Weisenberger, Clayton K. Collings, Sumeet Syan, Brian Hu, Mihir Desai, Monish Aron, Vinay Duddalwar, Inderbir Gill, Gangning Liang, Kimberly D. Siegmund

**Affiliations:** ^1^ Department of Urology, Norris Comprehensive Cancer Center, USC Keck School of Medicine, University of Southern California, Los Angeles, CA, USA; ^2^ Department of Preventive Medicine, Norris Comprehensive Cancer Center, USC Keck School of Medicine, University of Southern California, Los Angeles, CA, USA; ^3^ Department of Pathology, Norris Comprehensive Cancer Center, USC Keck School of Medicine, University of Southern California, Los Angeles, CA, USA; ^4^ Department of Biochemistry and Molecular Biology, Norris Comprehensive Cancer Center, USC Keck School of Medicine, University of Southern California, Los Angeles, CA, USA; ^5^ Department of Urology, Loma Linda University, Loma Linda, CA, USA; ^6^ Department of Radiology, Norris Comprehensive Cancer Center, USC Keck School of Medicine, University of Southern California, Los Angeles, CA, USA

**Keywords:** small renal mass, tumor classification, DNA methylation, kidney cancer

## Abstract

**Purpose:**

The clinical management of small renal masses (SRMs) is challenging since the current methods for distinguishing between benign masses and malignant renal cell carcinomas (RCCs) are frequently inaccurate or inconclusive. In addition, renal cancer subtypes also have different treatments and outcomes. High false negative rates increase the risk of cancer progression and indeterminate diagnoses result in unnecessary and potentially morbid surgical procedures.

**Experimental Design:**

We built a predictive classification model for kidney tumors using 697 DNA methylation profiles from six different subgroups: clear cell, papillary and chromophobe RCC, benign angiomylolipomas, oncocytomas, and normal kidney tissues. Furthermore, the DNA methylation-dependent classifier has been validated in 272 *ex vivo* needle biopsy samples from 100 renal masses (71% SRMs).

**Results:**

In general, the results were highly reproducible (89%, *n*=70) in predicting identical malignant subtypes from biopsies. Overall, 98% of adjacent-normals (*n*=102) were correctly classified as normal, while 92% of tumors (*n*=71) were correctly classified malignant and 86% of benign (*n*=29) were correctly classified benign by this classification model.

**Conclusions:**

Overall, this study provides molecular-based support for using routine needle biopsies to determine tumor classification of SRMs and support the clinical decision-making.

## INTRODUCTION

It is estimated that 62,700 new cases of renal cancer will be diagnosed in 2016 [1]. The incidence in the US has increased significantly over the past 10 years [2] due to increased use of abdominal imaging. However, although the incidence of renal cell carcinoma (RCC) is increasing, the mortality from this disease has not increased proportionately [1]. This is primarily attributed to the increased detection of localized small renal masses (SRMs), which are classified as tumors measuring < 4 cm in diameter and account for 48-66% of new kidney cancers [3]. In addition, 30% of SRMs are benign [4] and many SRMs having a low malignant potential. This is concerning as it has led to over diagnosis and over treatment for indolent lesions [5]. Nearly 65% of all renal masses are diagnosed when they are localized, and it has been shown that the incidence of benign pathology is inversely related to tumor size (i.e. a decrease in renal mass size increases the frequency of benign pathology) [6]. Current imaging techniques alone are unable to definitively distinguish benign from malignant pathologies [7]. Despite this, the majority of SRMs are still being treated without a pretreatment diagnostic biopsy, causing significant unnecessary morbidity to patients. Thus, renal tumor biopsies have the potential to assist in both the histological assessment and management of patients [3].

While radiologic imaging provides clues as to the pathology of the mass, incidental non-neoplastic findings such as trauma, infection, hemorrhage, infections, and cysts have radiographic features that occasionally are from those of the spectrum of renal carcinomas [7]. Furthermore, malignant and benign lesions appear to grow at similar rates, therefore this parameter cannot accurately identify malignant lesions requiring early intervention [8]. Currently, needle biopsies have been used along with radiologic assessment to evaluate SRMs, however, the applicability and the diagnostic and predictive accuracy of needle biopsy remain in question [9–11]. The accuracy of needle biopsy in distinguishing benign from malignant lesions ranges from 73-94%, but in SRMs, the needle biopsies have lower sensitivity, negative predictive value, and a high rate of false negativity [11].

It has been postulated that combining histological results with molecular markers can improve the sensitivity of needle biopsies [12]. While mRNA and protein-based markers are promising, in the SRM clinical scenario, the small amount of tissue available from the needle biopsy, sample stability issues, and the associated costs for subsequent analysis present significant challenges that make these markers burdensome choices.

DNA methylation alterations are among the first changes to occur in the process of tumorigenesis [13]. Because of this, it is likely that they will be present in the majority of tumors, as well as in less aggressive malignancies. Furthermore, they are easily detected in needle biopsy samples. DNA methylation is a stable modification from a stable DNA molecule, and therefore is less likely to be degraded in clinical samples. At the same time, PCR-based approaches allow for the analysis of DNA methylation using a very small sample with low costs. In fact, DNA methylation markers are currently being utilized to detect tumors in serum and urine sediments [14–17]. The fact that DNA methylation changes occur in RCC [18, 19] coupled with the ease of its detection, warrants further investigation to determine the applicability of utilizing DNA methylation markers to improve the accuracy of needle biopsies in SRMs in a clinical setting.

In this study, we built a classification model to predict subtypes of renal tumor that include benign and malignant. For subtypes of malignant tumors we have taken advantage of available DNA methylation data from The Cancer Genome Atlas (TCGA), and for subtypes of benign pathology we generated DNA methylation data from a new set of formalin-fixed paraffin embedded (FFPE) tumors. Finally, we applied the classifier to predict both the malignancy and tissue subtype on an independent data set of 272 *ex vivo* biopsies from 100 RMs (71 renal masses were SRMs, defined as < 4 cm). Overall, we demonstrate that cancer- and benign-specific DNA methylation data can be used as subtype-specific RCC biomarkers in needle biopsy specimens, which have potential utility in clinical decision-making, especially in SRMs.

## RESULTS

### Development of a DNA methylation classifier to subtype kidney tumors

RCC and its subtypes (clear cell, papillary and chromophobe) account for about 90% of solid renal masses, with clear cell accounting for over 75%, while the remaining 10% are composed of other malignancies (sarcoma, lymphoma, carcinoid) and benign solid tumors (oncocytoma, angiomyolipoma) [20]. We built a classification model for kidney tumors using Illumina Infinium HumanMethylation450 (HM450) DNA methylation data from 697 tissues across six major subgroups: 283 clear cell, 81 papillary and 65 chromophobe RCC, 27 benign angiomylolipomas, 37 oncocytomas, and 204 normal kidney. DNA methylation data for the 429 malignant cancers and 204 adjacent normal kidney tissues were obtained from TCGA (Supplemental Table 1), and additional HM450 DNA methylation data were generated for 64 benign tumors from FFPE microdissected tumor samples collected at the University of Southern California. The average size of the benign tumors was 3.4 cm, with 72% qualifying as small renal mass ( < 4cm).

**Figure 1:**
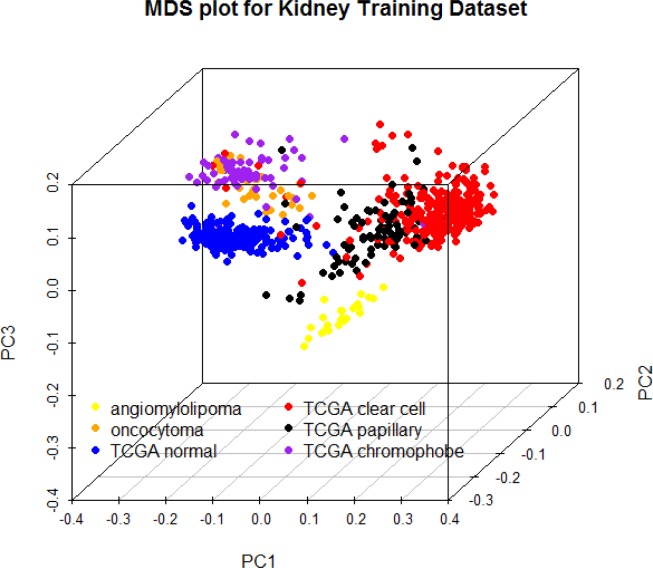
Multidimensional scaling plot of 697 training samples using the 500 features with greatest median absolute deviation

**Figure 2:**
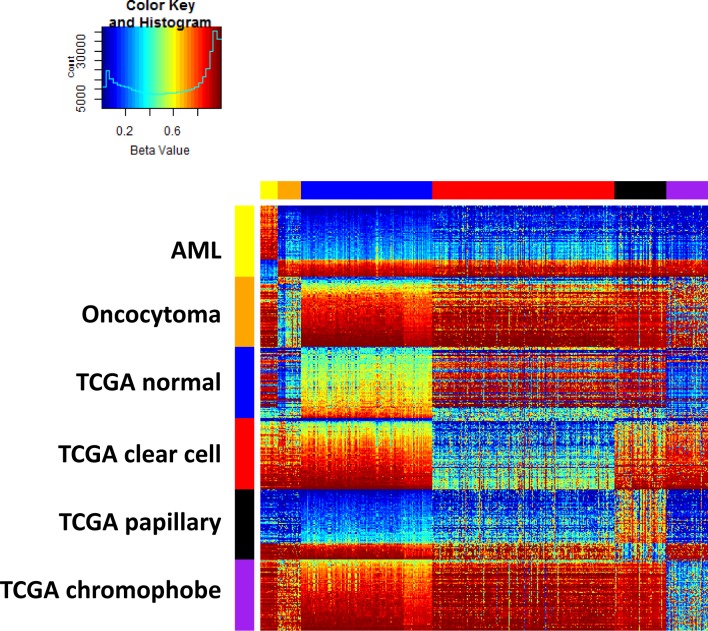
Training data set heatmap of 600 differentially methylated features (rows) in 697 kidney samples (columns) Columns are ordered by tissue subtype, and rows are ordered by sets of predictive features. Within each feature set, rows are ordered by average DNA methylation level in normal kidney.

A multidimensional scaling plot of the 697 training samples shows clustering of normal kidney and well-defined tumor subtypes (Figure 1). Angiomylolipomas (AML) form a distinct subgroup, oncocytomas and chromophobe RCCs cluster adjacent to one another, and clear cell and papillary RCCs cluster further away, indicative of unique DNA methylation profiles. For each subgroup, we selected the 100 CpG features with greatest separation of that subtype from all others, and combined all the lists. Interestingly, the six lists of features were unique and non-overlapping. Figure 2 shows an ordered heatmap of the training samples for the 600 selected CpG features (Supplemental Table 2). Whereas the majority of loci predictive of normal kidney have intermediate DNA methylation levels, they were decreased in oncocytomas and chromophobe RCCs and increased in AML (benign) and clear cell and papillary RCCs. The majority of loci predictive for a single tumor subtype showed consistent increases or consistent decreases when compared to the other subtypes.

The selected features for all subgroups were enriched with features outside UCSC CpG islands, shelfs and shores, with greater than 2-fold enrichment for chromophobe RCCs and benign oncocytomas (70% and 73% *vs* 32% reference) (Supplemental Figure 1). Enhancers were enriched 1.9-fold in AML and more than 2-fold in malignant tumors, normal kidney and oncocytomas. DNaseI hypersensitive sites showed the greatest variation in enrichment, with chromophobe RCC showing 4.5-fold depletion while AML, papillary RCC and normal kidney showed a 1.7-fold enrichment. This finding suggests that alterations of DNA methylation in the tumor subtypes mainly happened in enhancers but not promoter regions.

Furthermore, we built a multi-group classifier to predict tissue subtype, using an L_1_-penalty to reduce the DNA methylation feature set. The six groups were modeled using six equations, with each equation estimating the probability a sample belonged to one of the six groups and the sum of six probabilities equaling one. The final models used a combination of 59 variables: 2 for angiomylolipomas, 9 for oncocytomas, 11 for normal kidney, 13 for clear cell carcinomas, 14 for papillary and 10 for chromophobe RCC, with each model only selecting features from the subgroup-specific list (Supplemental Table 3). The classifier had 99.3% sensitivity and 99.6% specificity for the training data, detecting malignancy in 426 out of 429 cancers. Tumor subtype was predicted correctly in 95% of the training samples (407/429 malignant and 61/64 benign) (Supplemental Figure 2, Supplemental Table 4).

### Using *ex vivo* needle biopsies to validate the developed classification model

**Figure 3:**
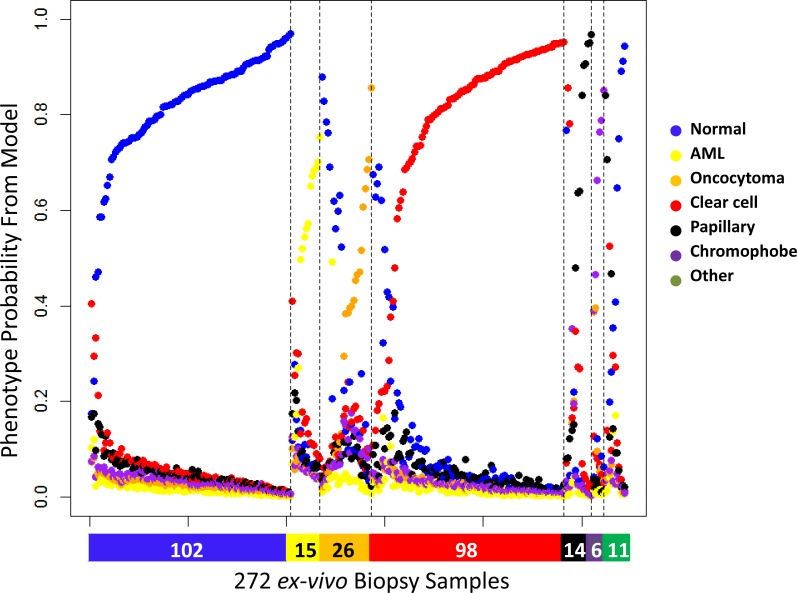
Six predicted probabilities for 272 *ex vivo* needle biopsy samples (102 normal kidney, 15 AML, 26 oncocytoma, 98 clear cell, 14 papillary, 6 chromophobe, 10 other benign, 1 other malignant) Color bar at the bottom denotes the diagnosis by pathology (blue: normal, yellow: AML, orange: oncocytoma, red: clear cell, black: papillary, purple: chromophobe, green: other). The probabilities are ordered by subgroup and the probability the sample is assigned to the correct subgroup.

We obtained 272 *ex vivo* needle biopsy samples from 100 renal masses after nephrectomy (partial or total) at USC. Based on pathology reports, there were 71 malignant RMs and 29 benign RMs. Seventy of these RMs were SRM with a clinical tumor size that was < 4 cm on radiologic imaging (Table 1). Consistent with other reports, the fraction of benign tumors was greater in the SRMs than in masses > 4cm (31% in SMRs *vs*. 24% in larger RMs). In general, three core biopsies were obtained from each patient: one from adjacent-normal tissue and two from the intact specimen using an 18-gauge side-cutting needle loaded on an automated biopsy gun. However, these numbers varied based on the availability of specimens across the patient set. For some *ex vivo* specimens, we only obtained one tumor needle biopsy. Figure 3 shows the prediction probabilities for the six phenotypes using HM450 DNA methylation data from these 272 *ex vivo* needle biopsies. The probabilities were plotted for the six groups, the color bar at the bottom indicating the corresponding diagnosis from the pathologist. The maximum probability for each sample represents the predicted phenotype. Malignancy status was correctly predicted in 93% of samples, (86% of papillary, 91% of clear cell, 100% of chromophobe, 98% of normal kidney, 100% of oncocytoma, 80% of AML, and 70% of other benign tumors) (Table 2). Subtype was correctly estimated in 85% of samples (range: 58%-100%).

**Table 1:** Clinical and pathological characteristics of samples included in the analysis

Patient (N)	100
Median age, years (Range)	65 (21-87)
Gender (%) Male Female	61.4% 38.6%
Median BMI, kg/m2 (Range)	27.7 (16.9-47.1)
Median clinical tumor size, cm (Range)	3 (1.0-10)
Mode of presentation (%) Incidental Symptomatic	97% 3%
Surgical treatment (%) Partial Nephrectomy Radical Nephrectomy	98% 2%
Median pathological tumor size, cm (Range)	2.7 (1.0-9.5)
Final diagnosis (%) Benign lesion Malignant lesion	27% 73%
pT Staging (%) pT1a pT1b pT2a pT3a pT3b	70.8 22.2 2.8 4.2 0
Lymph node involvement (%) pN0/Nx pN+	99% 1%
Distant metastasis (%) Absent Present	100% 0%

**Table 2:** Validation of 272 *ex vivo* needle biopsies (100 patients)

	Non-Malignant			Malignant		
	Benign^$^	Oncocytoma	Normal	Clear Cell	Papillary	Chromophobe
**Based on Biopsy (*N* = 272)**						
Ex Vivo Biopsy (N)	26	26	102	98	14	6
Correctly Predicted Subtype (N, %)	11 (73%)*	15 (58%)	100 (98%)	89 (91%)	9 (64%)	6 (100%)
Correctly Predicted Non-Malignant or Malignant (N, %)	12 (80%)*	26 (100%)	100 (98%)	89 (91%)	12 (86%)	6 (100%)
**Based on Tumors (*N* = 100)**						
Tumors (N)	13	16	-	59	8	3
Correctly Predicted Subtype (N, %)^1^	6 (75%)*	7 (44%)	-	53 (90%)	5 (63%)	3 (100%)
Correctly Predicted Non-Malignant or Malignant (N, %)	6 (75%)*	16 (100%)	-	54 (92%)	7 (88%)	3 (100%)

$ - consists of angiomyolipoma and other uncommon non-malignant lesions (i.e. capillary hemangioma, renal tubular hyperplasia, etc.)

^1^ - patient assigned subtype of biopsy with maximum posterior probability

* - prediction only of angiomyolipoma (N = 15 ex vivo samples, N = 8 tumors)

Classification error was evaluated as a function of the predicted probabilities. Entropy, the sum of p×log(p) for the six predictive probabilities p, captured classification uncertainty, with higher entropy for samples with more intermediate probability estimates and lower entropy for samples with greater discrimination in their probability estimates. Entropy varied by tumor subtype with benign AML and oncocytoma showing greater entropy compared to malignant tumors (Supplemental Figure 3). Not surprisingly, the entropy was also higher among samples predicted incorrectly than among those predicted correctly. Although 21 out of 59 features (36%) in our prediction model show age-related DNA methylation in normal kidney (Spearman *p* < 0.05), there was no significant association between age and entropy or age and samples predicted incorrectly. Seventy-two percent of samples had a maximum probability above 0.70. Malignancy was correctly estimated in 98% and subtype in 96% of this high-confidence sample subset.

Out of the 100 tumors studied, 70 had DNA methylation data from two needle biopsies. The prediction based on multiple needle biopsies assigned an individual tumor to be malignant if the needle biopsy results for either measurement was malignant. Each sample was assigned the subtype from the needle biopsy with the highest probability estimate. In general, the results were highly reproducible with 62 of 70 tumors (89%) predicting identical subtypes from both biopsies. However, seven of the 62 concordant pairs (11%) were incorrectly predicted as normal kidney, of which two were missed malignant tumors (2 clear cell RCC), 3 ‘other’ benign, and 2 oncocytomas. Three malignant tumors with discordant needle biopsy results were correctly predicted as malignant when using two needle biopsies (2 clear cell, 1 papillary RCC). Overall, the sensitivity estimates at the tumor level reflected similar estimates at the sample level (Table 2). Sixty-five out of 71 (92%) tumors were correctly classified as malignant and 25 of 29 (86%) were correctly classified as benign. Importantly, the sensitivity and specificity did not vary by tumor size ( < = 4cm). In SRM the sensitivity was 90% (44 out of 49) and specificity 86% (19 out of 22).

Taken together, the high specificity and sensitivity to predict not only benign and malignant in SRMs but also the more detailed subtypes holds great promise for our DNA methylation classification model to develop into a DNA methylation-based assay for needle biopsy samples.

## DISCUSSION

Treatment decision making for SRMs is an increasingly frequent and challenging clinical problem. The management of SRMs first requires accurate characterization, and then the options for treatment consist of active surveillance, surgical removal, or *in situ* ablation. This decision of the best treatment modality is based on clinical assessment of patient comorbidities and tumor characteristics. SRMs are represented by a heterogeneous group of benign and malignant histologic entities, with a range of biologic and clinical behaviors. However, the assessment of tumor malignancy generally relies on its size, shape, profile, as well as tissue enhancement on multiphasic computed tomography (CT) and magnetic resonance imaging (MRI). The use of renal tumor biopsies to obtain pathologic information to guide treatment decisions has been traditionally reserved for very selected cases of SRMs [21]. Before the advent of biologic-targeted therapies, there was also limited interest in the histologic characterization of advanced and metastatic renal tumors.

Needle biopsies have demonstrated an ability to improve kidney tissue selection while maintaining a low complication rate. However, a key limitation of needle biopsy is its high rate of false negative results [22], as a sensitivity of 80% has been previously reported [23]. Using molecular markers is one potential way to increase both sensitivity and specificity, as demonstrated by our approach. Our hypothesis is that by incorporating a DNA methylation assay derived from needle biopsies, patients will be placed into more appropriate treatment protocols. This could potentially reduce invasive and morbid SRM treatments, especially in the elderly or in patients with benign diseases. In fact, the American Urological Association recommendations for the management of localized renal tumors states the study of molecular and genetic profiling on percutaneous renal tumor biopsies as a research priority (https://www.auanet.org/education/guidelines/renal-mass.cfm).

There have been previous reports that have found DNA methylation biomarkers specific for clear cell but provide limited data on other subtypes of RCC and benign tumors [24–26]. In our study, we provide detailed methylation profiling of subtypes of benign phenotypes, which we developed from microdissection of FFPE tissue from kidney cases resected at the University of Southern California. Furthermore, we not only established specific DNA methylation panel of subtypes of RCC and benign tumors but also further validated our methylation panel from a core needle biopsy. It has been reported previously that only 17% of all benign renal masses were correctly defined on radiologic imaging [27]. The addition of methylation profiles for the subtypes of benign SRMs *via* needle biopsy allows for the improved detection of benign pathologies of SRMs and may theoretically reduce patient morbidity.

A needle biopsy collects a very small amount of tissue; thus our panel for needle biopsy is novel and has clinical applicability. Needle biopsies of SRMs can be completed pre-operatively for diagnostic assessment. As previously stated, needle biopsies have high false negative results, along with diagnostic accuracy as low as 80% [28]. While the causes of those issues are multiple, currently the best way to overcome those is by repeated biopsy; however when multiple biopsies were taken by different techniques and different sizes, the diagnosis was not conclusive in all biopsies taken in the majority of cases (54.7%) [23]. This demonstrates a significant operator error that we believe DNA methylation may aid in minimizing. However, a repeat biopsy provides many difficulties as it causes many side-effects such as increased morbidity on the patient for another procedure, increased healthcare costs, and it becomes an even more difficult procedure for the urologist, especially in SRM. This would lead to the notion that the addition of a DNA methylation profile that can improve diagnosis of a SRM can lead to reduced patient morbidity by potentially preventing unnecessary surgical interventions.

To identify candidate markers that are differentially methylated in RCC and build a classification model, we have taken advantage of the TCGA database [29–31], which contains Illumina Infinium HM450 DNA methylation data for 429 malignant RCCs and 204 normal-adjacent tissues. Although most of these tumors were too large to be classified as SRM (median clinical tumor size is 5.54 cm for clear cell renal carcinomas, 9.6 cm for chromophobe renal carcinoma, 5.35 cm for papillary renal carcinoma) [29–31], the large sample size allowed us to build a prediction model that we later validated using *ex vivo* needle biopsies from an independent data set that included 71 SRMs. Importantly, we found that the sensitivity and specificity of diagnosing malignant subtype did not vary with tumor size ( < = 4cm *vs* > 4cm). This is consistent with reports that DNA methylation changes are among the first alterations to occur during tumorigenesis [13] and suggests that changes identified from large renal masses are predictive in small renal masses. However, this warrants further validation in a larger sample set.

The addition of 64 non-malignant tumor samples from our laboratory further allowed us to test whether there are specific patterns in the non-malignant tumors and their subtypes. These data strongly suggest that differential DNA methylation patterns exist not only between non-malignant and malignant tissues, but also among tumor subtypes. In particular, we found that subtype-specific markers predictive of angiomyolipoma that differed from those predictive of oncocytoma, supporting the further study of these subtypes in larger sample sizes to improve the predictive ability for these major benign subclasses. Also, confirming results from a recent report [32], chromophobe RCC appears more similar to benign oncocytoma than the other malignant papillary and clear cell tumors, supporting our hypothesis that cancer-specific DNA methylation can be used as subtype-specific renal cancer biomarkers. In further support of this, the six sets of probes we used to predict each subtype are indeed non-overlapping, allowing for the identification of subtypes using DNA methylation data.

Normal kidney tissues was predicted with high specificity using DNA methylation data. Although normal kidney from healthy individuals was not available for study, 27% of the normal kidney in our test set came from cancer-free individuals (patients with benign lesion only). All of the normal kidney from this patient subset was correctly identified as normal kidney. Interestingly, the two normal kidney samples that were incorrectly classified as clear cell carcinomas came from patients with clear cell tumors, suggesting that the biopsy might have contained tumor cells from the patient. We also found the reverse, in which clear cell tumors were incorrectly classified as normal. However, these classification probabilities were greater than 20% for being clear cell, suggesting that the biopsy may not have captured a sufficient number of malignant cells. This suggests that the classifier accurately reflects cell mixtures based on the probabilities it assigns to the individual subgroups.

The highest error rates occurred for the benign tumor subtypes. The benign tumors most likely to be overcalled as malignant were those from subtypes that were too rare to be represented in our training dataset [12]. The poor performance for AML and oncocytomas might be a result of the limited sample numbers (27 AML and 37 oncocytomas) for these subtypes and indicate a need to include more samples in future studies in order to establish a better separation pattern.

In summary, this validation of our kidney tissue specific DNA methylation panel in *ex vivo* needle biopsy illustrates a proof-of-principle approach. The success of this approach could lead to future clinic trials on patient *in vivo* biopsy. Our data demonstrate that differential DNA methylation patterns exist not only between benign and malignant tissues, but also between tumor subtypes. These results fully support our hypothesis that cancer-specific DNA methylation can be used as subtype-specific RCC biomarkers. This DNA methylation classification model could allow for improved clinical management of RCC patients, in which unnecessary surgical procedures would be minimized for patients with benign lesions, thereby reducing patient-associated morbidity/mortality. Moreover, malignant lesions and their subtypes can be identified earlier, thus decreasing unnecessary radiation exposure from serial imaging and increasing the chance of preserving renal function.

## MATERIALS AND METHODS

### Patient material, samples, and marking

In a prospectively-collected institutional review board (IRB)-approved database, *ex vivo* samples were collected from resected kidney tissue retrieved immediately post-operative. For each surgical specimen, three doublet biopsies were taken: two doublets in the mass, and one doublet in normal kidney parenchyma adjacent to the mass. One sample from each doublet was used for H&E preparation, and the other sample was used for DNA methylation analysis. DNA extraction from the biopsy allotted a median amount (range) of 4.6 (1.4-38.9) ug of DNA. FFPE-microdissected samples of 64 benign tumors were collected from our institution's IRB-approved renal tissue database. A trained pathologist reviewed each prospective kidney case and the block that contained the most pure pathology was selected for microdissection.

Training data include a total of 697 kidney samples consisting of 6 subtypes: 283 clear cell carcinomas, 81 papillary carcinomas, 65 chromophobe, 27 angiomylolipomas, 37 oncocytomas, and 204 normal kidney. HM450 profiles for the malignant cancers and normal kidney tissues were downloaded from the TCGA data portal (https://tcga-data.nci.nih.gov/tcga/), and supplemental HM450 DNA methylation profiles were generated for the FFPE-microdissected samples of 64 benign tumors collected at USC. A testing dataset comprised of 272 *ex vivo* needle biopsy samples collected from 100 patients after nephrectomy (partial or total) at USC. The study population consisted of the first 100 consecutive patients who consented to participate in the study. The 272 *ex vivo* samples included 98 clear cell, 14 papillary, 6 chromophobe, 102 normal kidney, 15 angiomylolipoma, 26 oncocytoma, 10 other benign and 1 other malignant. Seventy tumors had data from two needle biopsies.

### DNA methylation profiling

Genomic DNA (200-500ng) from each FFPE sample was treated with sodium bisulfite and recovered using the Zymo EZ DNA methylation kit (Zymo Research) according to the manufacturer's specifications and eluted in 10μl volume. An aliquot (1μl) was removed for MethyLight-based quality control testing of bisulfite conversion completeness and the amount of bisulfite converted DNA available for the Illumina Infinium HM450 DNA methylation assay [33]. All samples passed the QC tests and were then repaired using the Illumina Restoration solution as described by the manufacturer. Each sample was then processed using the Infinium DNA methylation assay data production pipeline [34].

All HM450 profiles were generated at the USC Molecular Genomics Core Facility. All data were processed from IDAT files using the *minfi* and *wateRmelon* packages in Bioconductor. DNA methylation is reported as Beta values, the proportion of methylated target. Measures are corrected for background intensity, dye bias and typeI/typeII design bias using ‘noob’ followed by BMIQ. Beta values from features with low signal intensity were assigned as missing and samples with more than 5% features missing were excluded. One sample was excluded from the test set for this reason. We applied the feature filter from TCGA omitting features due to SNPs, repetitive regions, or targeting CpH sites, also filtering features mapping to X or Y chromosomes. Features containing missing values in either training or testing dataset are excluded, leaving a final data set of 351,124 features.

### Pre-selecting DNA methylation markers

We used the training data to select *a priori* a list of 100 features for each of the six renal tissue subtypes as a function of their differences in group means. Specifically, for each subtype, we ranked the features on the smallest difference in average Beta value between the given subtype and each remaining subtype. Then, the top 100 features with the *largest* minimum absolute differences are selected. No feature was selected twice, resulting in a combined set of 600 features. These 600 features are displayed in a heatmap and used for training the classification model (Figure 2).

### MDS plot and heatmap

A multidimensional scaling (MDS) plot of the 500 features with greatest median absolute deviation was created using the *limma* package. The heatmap shows a supervised clustering of the samples in the training data set for the 600 differentially-methylated CpG features. The columns represent samples and the rows represent predictive features, each ordered by group as follows: *ex vivo* angiomyolipoma, *ex vivo* oncocytoma, TCGA normal kidney, TCGA clear cell, TCGA papillary, and TCGA chromophobe RCCs.

### L_1_-penalized classification model

To predict tissue subtype we fit the L_1_-penalized multinomial logistic regression model using the *GLMnet* package in the R programming language. We provided as input the 600 features on 697 training samples, and performed 10-fold cross-validation to select the penalty parameter and reduced feature set. We tested the model on 272 *ex vivo* needle biopsy samples collected from 100 tumors after nephrectomy (partial or total) at USC.

The output of the GLMnet model is probabilities of belonging to each subgroup, as a function of the DNA methylation values of the selected features. For each sample, the probabilities for the six renal tissue subtypes sum to one and we assign each sample to the subgroup with the highest predicted probability. Classification error rates are evaluated using pathology as the gold standard. Error rates were assessed for two classifications: (1) discriminating malignant *vs*. non-malignant and (2) discriminating the six tissue subgroups. For the classification of malignant/non-malignant, clear cell, papillary, and chromophobic RCC are classified as malignant, and AML, oncocytoma and normal kidney as non-malignant.

### Ethics approval and consent to participate

The study was conducted after USC IRB approval and following written informed consent from all patients. HS-12-00147

### Availability of data and materials

The datasets supporting the conclusions of this article are available in the Open Science Framework repository, DOI 10.17605/OSF.IO/Y8BH2 | ARK c7605/osf.io/y8bh2 at https://osf.io/y8bh2/.

## SUPPLEMENTARY FIGURES










