# Supplementary material for: Improving needle biopsy accuracy in small renal mass using tumor-specific DNA methylation markers
Source: Oncotarget. 2016 Sep 27;8(3):5439–48. doi: 10.18632/oncotarget.12276 (PMC5354921; doi:10.18632/oncotarget.12276)
Supplement: Supplementary file 1 [file oncotarget-08-5439-s001.pdf]

Improving needle biopsy accuracy in small renal mass using tumor-specific DNA methylation markers

Supplementary Material

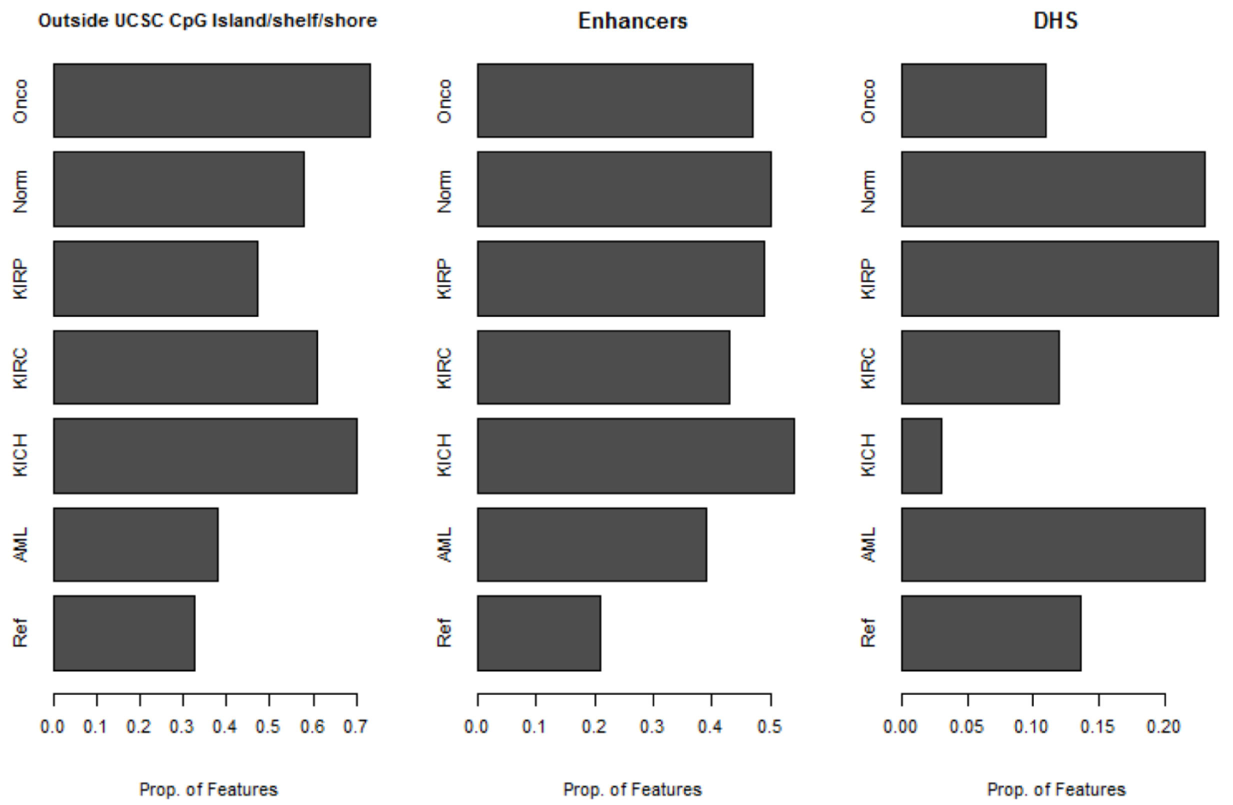

Supplemental Figure 1. Fraction of 100 subtype-predictive features showing the attribute of interest. Reference is the 351124 features that remained after filtering.

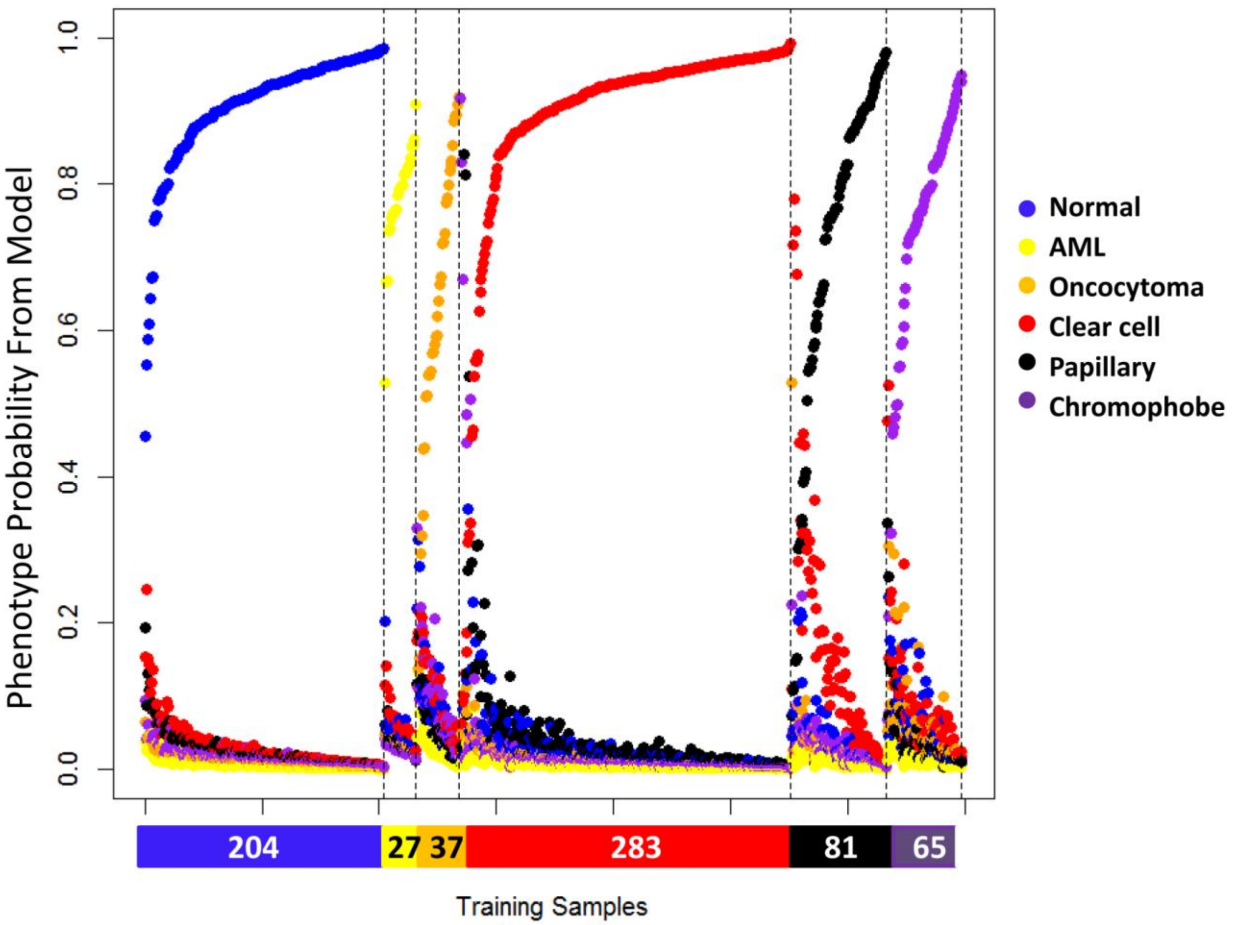

Supplemental Figure 2. Six predicted probabilities for 697 kidney training samples (283 clear cell carcinomas, 81 papillary carcinomas, 65 chromophobe, 27 angiomylolipomas, 37 oncocytomas, and 204 normal kidney). Color bar at the bottom denotes the subgroup (blue: TCGA normal, yellow: AML, orange: oncocytoma, red: TCGA clear cell, black: TCGA papillary, purple: TCGA chromophobe).

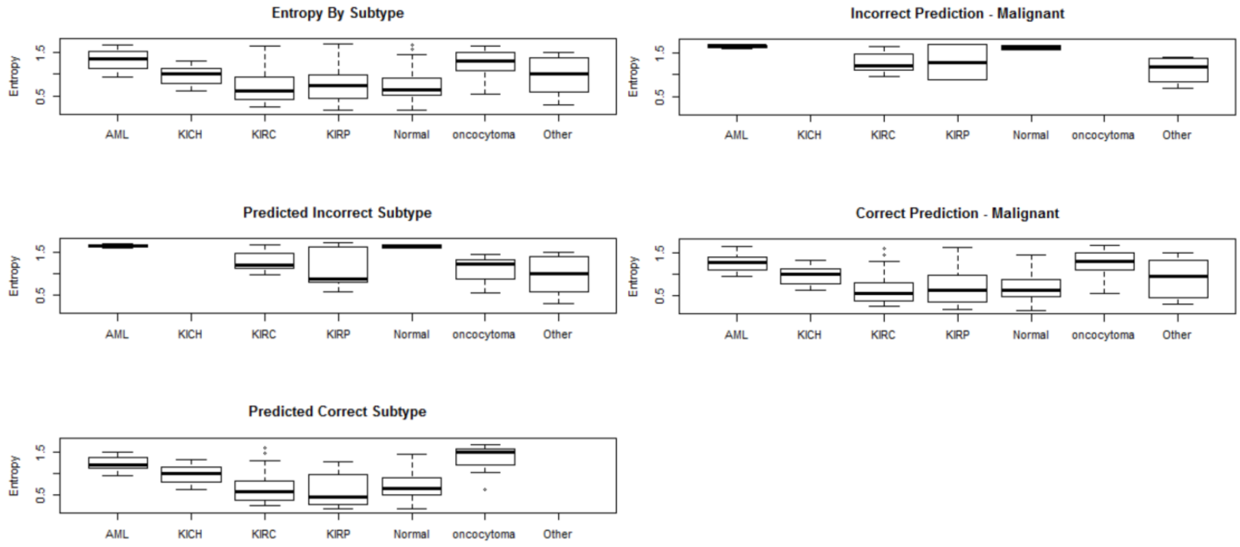

Supplemental Figure 3. Boxplots of the entropy for each sample ($-\Sigma_i\ p_i*\ln(p_i)$) where p_i is the estimated probability of group i, i=1,…,6). Top left is overall, Left middle is for samples with subtype incorrectly predicted, left bottom is for samples with subtype correctly predicted. Top Right is for samples with malignancy incorrectly predicted and Right middle is for samples with malignancy correctly predicted.
